# Use of existing hydrographic infrastructure to forecast the environmental spawning conditions for Eastern Baltic cod

**DOI:** 10.1371/journal.pone.0196477

**Published:** 2018-05-16

**Authors:** Burkhard von Dewitz, Susanne Tamm, Katharina Höflich, Rüdiger Voss, Hans-Harald Hinrichsen

**Affiliations:** 1 GEOMAR Helmholtz Centre for Ocean Research Kiel, Kiel, Germany; 2 Federal Maritime and Hydrography Agency, Hamburg, Germany; 3 Sustainable Fishery, Department of Economy, University of Kiel, Kiel, Germany; Technical University of Denmark, DENMARK

## Abstract

The semi-enclosed nature and estuarine characteristics, together with its strongly alternating bathymetry, make the Baltic Sea prone to much stronger interannual variations in the abiotic environment, than other spawning habitats of Atlantic cod (*Gadus morhua*). Processes determining salinity and oxygen conditions in the basins are influenced both by long term gradual climate change, e.g. global warming, but also by short-term meteorological variations and events. Specifically one main factor influencing cod spawning conditions, the advection of highly saline and well-oxygenated water masses from the North Sea, is observed in irregular frequencies and causes strong interannual variations in stock productivity. This study investigates the possibility to use the available hydrographic process knowledge to predict the annual spawning conditions for Eastern Baltic cod in its most important spawning ground, the Bornholm Basin, only by salinity measurements from a specific location in the western Baltic. Such a prediction could serve as an environmental early warning indicator to inform stock assessment and management. Here we used a hydrodynamic model to hindcast hydrographic property fields for the last 40+ years. High and significant correlations were found for months early in the year between the 33m salinity level in the Arkona Basin and the oxygen-dependent cod spawning environment in the Bornholm Basin. Direct prediction of the Eastern Baltic cod egg survival in the Bornholm Basin based on salinity values in the Arkona Basin at the 33 m depth level is shown to be possible for eggs spawned by mid-age and young females, which currently predominate the stock structure. We recommend to routinely perform short-term predictions of the Eastern Baltic cod spawning environment, in order to generate environmental information highly relevant for stock dynamics. Our statistical approach offers the opportunity to make best use of permanently existing infrastructure in the western Baltic to timely provide scientific knowledge on the spawning conditions of Eastern Baltic cod. Furthermore it could be a tool to assist ecosystem-based fisheries management with a cost-effective implementation by including the short term predictions as a simple indicator in the annual assessments.

## Introduction

Climate and anthropogenic forcing induce a highly variable abiotic environment in the Baltic Sea [1]. All major Baltic fish populations are affected by the environmental variability both with respect to growth and recruitment. For example, the growth rates of herring and sprat diminish with reduced salinity in the eastern and northern part of the Baltic [2,3]. It has also been indicated that the recruitment of sprat in the entire Baltic is significantly correlated to temperature [4] as well as to wind-driven larval transport patterns [5]. Abiotic variations translate over physiological processes and food web dynamics into changes in fish stock productivity [6], and hence challenge sustainable fisheries management [7]. However, recruitment estimates in the assessment procedure currently employed do not include any environmental factors, and hence neglect these processes [8]. In the Baltic the fishing industry as well as consumer representatives have expressed a major interest in relative year-to-year stability of catch options, determining the total amount of permitted catch per species [9,10]. Inclusion of environmental factors in the assessments with short-term forecasts of environmental conditions, possibly enhancing the recruitment estimations for the coming year might be a way forward towards more environmental orientated and stable advice on sustainable catches.

**Fig 1:**
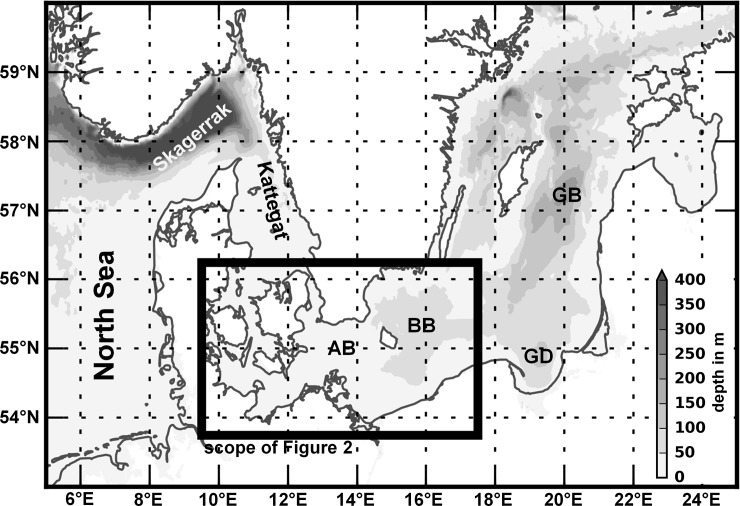
Overview of the Baltic Sea region. Main basins of the Baltic Sea are indicated in abbreviated form by AB (Arkona Basin), BB (Bornholm Basin), GD (Gdansk Deep) and GB (Gotland Basin). The black frame indicates the scope of the more detailed map in Fig 2. Bathymetric data ETOPO1 are taken from Amante and Eakins [18].

Here, we concentrate on Eastern Baltic cod, which is of high ecological as well as economic importance and is historically the third largest Atlantic cod stock[11]. The stock is distributed in the south-eastern Baltic Sea with the main spawning grounds being the Bornholm Basin, the Gdansk Deep and the Gotland Basin, and additionally it is sharing the Arkona Basin with the western Baltic cod stock [12] (Fig 1). Since the onset of sufficiently detailed records around 1966, the stock showed large fluctuations in stock size, growth, and especially recruitment [13] which have been shown to strongly depend on various environmental factors [13–15]. During the end of the 1980s the stock drastically declined [16] due to a combination of overfishing and the absence of major Baltic inflow events [17], resulting in bad environmental conditions, both in terms of sufficient salinity and oxygen conditions for reproduction in the sub-basins. Currently, the stock seems to be in a regime of comparatively low productivity with indications of an upward trend [15].

Recruitment is both related to egg production but also to egg survival probability depending on hydrographic conditions and predation [14,15]. Based on experimentally established thresholds of salinity, temperature and oxygen for egg survival, MacKenzie et al. [19]quantified the volume of water that permitted successful Eastern Baltic cod egg development, the reproductive volume. Spatial comparisons demonstrated extensive differences between the three main spawning areas, i.e. the Bornholm Basin, Gdansk Deep, and Gotland Basin (Fig 1). The reproductive volume was highly variable in the last 60 years but was found on generally low levels since 1980 [19]. In recent years, only the Bornholm Basin remained as a major spawning ground, due to regular occurrence of hypoxic conditions at the other two historic spawning sites [20].

**Fig 2:**
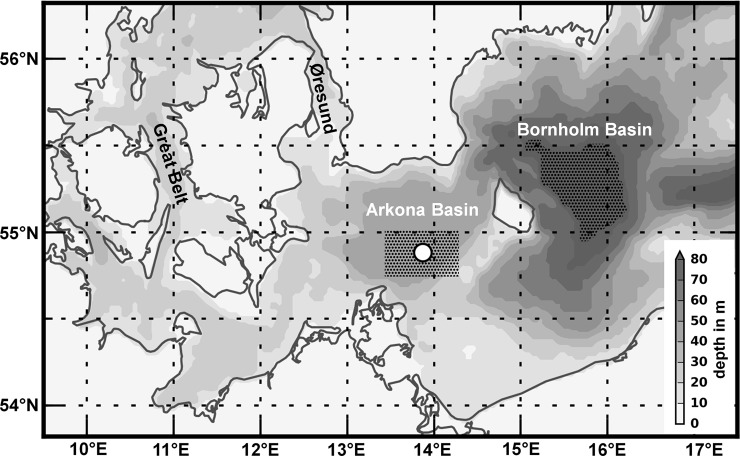
Detailed map of the south-western Baltic Sea. The white dot indicates the location of the observational platform in the Arkona Basin. Areas selected for vertically resolved hydrodynamic model data are depicted in shaded areas around the platform and additionally in the Bornholm Basin. Bathymetric data taken from the hydrodynamic Kiel Baltic Sea Ice-Ocean Model described in e.g.[26].

The main factor determining the dynamics of suitable water masses for cod reproduction in the Eastern Baltic Sea is the advection of highly saline and well-oxygenated water masses from the North Sea and Kattegat area (Fig 1). These water masses enter the central Baltic Sea through the Great Belt and the Øresund (Fig 2). Schinke and Matthäus [21] identified the occurrence of high atmospheric pressure associated with easterly winds followed by longer time periods of westerly gales over the North Atlantic and Europe, with only small fluctuations in direction, as principle major Baltic inflow mechanisms. Stigebrandt [22] showed that the inflowing water before entering the Bornholm Basin forms a pool in the deeper part of the Arkona Basin. In general, the inflowing saline water masses manifest as density-driven bottom gravity currents that entrain ambient surface water and interleave at levels of neutral buoyancy along the cascade of sub-basins[23]. But hydrodynamic modelling exercises performed by Krauß and Brügge [24] showed that further processes such as wind-induced return flows are important for the exchange of deep water masses between the sub-basins of the central Baltic Sea, highlighting the general complexity.

Major inflows into the Baltic usually take place in the period from August to April, and in 60% of the cases between November and January [25]. During recent decades larger inflow events were less frequent. Minor inflow events, however, were recorded regularly [26], which had a positive effect on the reproductive volume sustaining egg development in the Bornholm Basin. The improvements of the oxygen-related spawning conditions, however, often did not persist to the end of the spawning season in August, and the reproductive volume returned quickly again to low levels in the middle of the summer [27].

Standard advice on fishing opportunities and catch of Eastern Baltic cod is based on yearly stock assessments and short-term population forecasts, not including any environmental considerations [8,28]. A major obstacle preventing the use of environmental data in stock assessment is the necessity for in time and regularly available information, which can in most cases not be guaranteed on the basis of e.g. ship based investigations. Stock assessment efforts are usually started in March each year when environmental data of the current year are simply not yet available, because compiling and checking data is time consuming. Within the Baltic Sea collective data base for oceanographic measurements at ICES, data is usually available with a lag of 2 to 4 months. Furthermore it is collected during ship cruises, which are often prevented from operating by bad weather conditions and are performing measurements at specific time points and locations only. Therefore we examine in this study, the potential use of regular, standardized hydrographic measurements carried out on a permanently installed observational platform in the western Baltic as a short-term indicator to predict oxygen conditions during the spawning time in the Bornholm Basin.

To this end, we run a hydrodynamic ocean model setup to hindcast in detail the salinity conditions in the Arkona Basin and oxygen conditions in the Bornholm Basin for the last 40 years to be used for the analysis. Salinity measurements at the observational platform were only started from 2002 and actual measurement data, from e.g. the ICES Oceanographic data base, is also not available in a sufficient coverage of the last 40 years to make a comprehensive analysis. Therefore using the model data, we first investigate the general correlations between salinity conditions in the Arkona Basin (western Baltic Sea) and oxygen conditions in the Bornholm Basin. Furthermore we analyse the predominant development of the oxygen content in the Bornholm Basin during the spawning season and demonstrate that oxygen conditions generally decline after the inflow season, and that the oxygen condition for April, the beginning of the spawning season, is an excellent indicator for the egg survival probability during the entire annual reproductive cycle. Finally, we determine a functional relationship between salinity conditions in the Arkona Basin and oxygen conditions as well as cod egg survival probability in the Bornholm spawning ground for eggs spawned by different age-classes of female cod.

## Material & methods

### Measurement platform

The mentioned permanently installed measurement platform was newly deployed with a semi-submersible floating platform in 2002 by the Leibniz Institute for Baltic Sea Research, Warnemünde (IOW) on behalf of the Federal Maritime and Hydrographic Agency (BSH) and is also maintained by IOW on behalf of the BSH. It is situated in the Arkona Basin at the position of 54°53' N, 13°52' E (Fig 2) and is part of the automated German marine monitoring network in the North Sea and Baltic Sea (MARNET). The maintenance of the platform is scheduled for at least six visits per year. In case of malfunction or damages the number of visits is increased. At the beginning and at the end of the maintenance CTD measurements are conducted. These measurements are used to identify and correct data drifts.

The platform is equipped with a set of hydrographic sensors measuring temperature and salinity that are placed in 8 different depths between 2 and 43m (specifically 2, 5, 7, 16, 25, 33, 40 and 43 m). Two of which are also equipped with an oxygen sensor. The station transmits hourly real-time data ashore. This transmission uses Meteosat as well as the Global System for Mobile Communications (GSM). Data is undergoing a real time quality control that is agreed in the CMEMS in situ TAC for all European monitoring stations (CMEMS = Copernicus Marine Environment Monitoring Service; In situ TAC = in situ Thematic Assembly Center; A description of the quality control procedures can be downloaded from: http://marine.copernicus.eu/documents/QUID/CMEMS-INS-QUID-013-030-036.pdf). Data is made freely available under the Copernicus Service (for registered users) or by request from the BSH. It is also available in a delayed mode with an enhanced quality control. This enhanced quality control comprises of an additional comparison against a climatology and visual inspection, delaying the data availability by one month.

To include a succession of several inflow and stagnation periods in the analysis and with that produce more reliable functional relationships between the Arkona Basin salinity and the spawning conditions for Eastern Baltic cod in the Bornholm Basin, we aimed at using a dataset at least covering several decades. Therefore other sources of data than the Arkona Station platform established in 2002 were needed.

### Data source for the analyses

As a preliminary test in situ abiotic data taken from the ICES hydrological data base was downloaded (available at http://ocean.ices.dk/HydChem) [29], averaged with monthly resolution in 5m depth intervals and tested for their applicability in this study. Unfortunately especially for the winter period from November to February, available coverage was not sufficient. For the depth layer 30-35m within the area chosen around the platform (Fig 2), for example, data is available for the months December and January only in 10% and 30% of the years between 1971 and 2013, respectively. Moreover, in most cases when data is available, it consists of snapshots, i.e. only one or two data points per month. Reasons could be the prevailing weather conditions during this period and generally a higher interest in more biological active seasons. The spatially and temporally highly variable salinity environment in the Arkona Basin is not sufficiently represented by this data to perform a comprehensive analysis. The derived relationships could not be applied to hourly measured data from the Arkona observation platform. A solution to this problem is offered by using hydrographic data simulated by numerical ocean model setups, which were used instead for the analyses in this study. The advantage is the high spatial and temporal resolution, and results have been shown to be in very good agreement with field observations in terms of simulating average conditions and interannual variations. Lehmann et al. [26] showed that the mean and natural variability range is well captured by the model and stated that the correspondence between two individual CTD profiles and the profiles produced by the model for the same locations and time points were very high. The simulated conditions provide a “quasi-synoptic” view on the environmental dynamics at discrete locations and the compiled salinity and oxygen data sets can be used for a comprehensive analysis of characteristics of the ecosystem.

Therefore the basis for our analysis was chosen to be the hydrodynamic Kiel Baltic Sea Ice-Ocean Model (BSIOM) [30,31]. The horizontal resolution of the coupled sea ice–ocean model is at present 2.5 km. In the vertical 60 levels are specified, which enables the upper 100 m to be resolved into levels of 3 m thickness. The model domain comprises the Baltic Sea, Kattegat and Skagerrak. At the western boundary, a simplified model of the North Sea is connected to the Baltic model domain to provide characteristic North Sea water masses entering the domain. The oxygen conditions in the entire Baltic Sea are described by an oxygen consumption sub-model coupled to BSIOM [26]. It describes one pelagic, one benthic and one sediment oxygen sink, and consumption rates are calculated based on temperature and oxygen [32], where rates are higher at higher temperatures and rates are lower at lower oxygen levels. The coefficients of the oxygen model were originally developed for the transition region between North Sea and Baltic Sea. As primary production in the Baltic Sea is smaller, oxygen consumption rates are scaled for each sub-basin relative to the Kattegat and Belt Sea. Furthermore, to account for long-term changes in oxygen consumption from i.e. eutrophication, rates are further scaled to include trends in primary production [26].

The model is forced by low frequency sea level variations in the North Sea/Skagerrak region calculated from the BSI (Baltic Sea Index) [31,33]. For this study two model setups have been used that differ in the atmospheric forcing. The first setup is forced by the Swedish Meteorological and Hydrological Institute (SMHI) meteorological database (Lars Meuller, pers. comm.) which for the period 1970–2010 covers the whole Baltic drainage basin. The second setup is forced by ERA-Interim reanalysis fields [34] that allow with regular updates until the present for a simulation of the period since 1979. The first model setup was run for the period 1970–1979, the second for the period 1979–2015. Hydrographic profiles were extracted in daily resolution.

Two series of hydrographic profiles with information on salinity and oxygen were extracted from the model output. This was done by averaging each depth layer over the two areas shown in Fig 2, representing the area around the observational platform in the Arkona Basin and the part of the Bornholm Basin with a water depth >70m. The temporal resolution was decreased for the analyses to a monthly resolution. The resulting monthly profiles in the vertical model resolution of 3 m for both areas were used to investigate correlations between salinity in the Arkona Basin and oxygen in the Bornholm Basin (Analysis #1). The Bornholm Basin profiles were further averaged within two integrated depth layers below the halocline to test for a functional relationship for water depths relevant for cod reproduction (Analysis #3). Moreover the profiles for the Bornholm Basin were interpolated in regard to water density (egg buoyancy) to investigate the seasonal development of the spawning conditions (Analysis #2), as well as the functional relationship between salinity in the Arkona Basin and the spawning conditions at 3 specific density layers relevant for cod reproduction (Analysis#3).

### Arkona Basin salinity to Bornholm Basin oxygen correlations (Analysis #1)

We first focused on the correlations of salinity in the Arkona Basin with oxygen in the Bornholm Basin. Used were those time periods with the majority of Baltic inflow situations (November to January) extended by two months to March. In that way we included data up to the start of the Eastern Baltic cod spawning season in April [12,35,36]. To analyse large-scale influences of Baltic inflow events, all data of the Bornholm Basin below 50 meters was included and correlations were performed for lags up to 2 months for the period 1971–2015. The vertical resolution of the used profiles corresponded to the vertical resolution of the BSIOM of 3 m layers. Before applying cross-correlation statistics in between time series for each month and depth level, they were linearly detrended, checked for autocorrelation by the Ljung-Box [37] and Durbin-Watson tests [38]; and reduced to the residuals of an AR model fit of increasing order until the tests results were negative. In the following correlations in between the resulting time series of salinity and oxygen the strongest correlations were found for the 33m level in the Arkona Basin. The subsequent analyses were therefore based only on the Arkona Basin salinity at this depth level, and tested for a dependency of the environmental conditions within the Bornholm Basin on the salinity levels within the Arkona Basin (Analysis #3 and Example).

### Temporal development of the oxygen content in the Bornholm Basin during the spawning season (Analysis #2)

To investigate the seasonal oxygen development following the inflow period in the spawning habitat of cod, the model data was used to assemble monthly mean values of oxygen content in the Bornholm Basin on different buoyancy levels by interpolation in regard to density (1009, 1011, 1013 kg m^-3^, corresponding to a salinity of ca. 11, 13.5 and 16 at a temperature of around 5°C). These levels were chosen to represent the buoyancies of eggs of different female age categories: old (large), mid-age (medium size) and young (small) cod, and were based on the results described in Vallin and Nissling [39]. Oxygen time series at all buoyancy levels for April were compared to the time series found for August to determine the common direction of the seasonal oxygen development. Furthermore the integral of the oxygen concentration between May and August on all buoyancy levels was calculated for each year. With that, the dependency of the oxygen concentration during the entire spawning season on the initial value in April could be investigated. Tests were performed with linear correlation statistics between the mean value in April as independent variable and the integral from May to August as dependent variable. The 1013 kg m^-3^ density level did not exist in the Bornholm Basin in 15 of the 44 years due to overall low salinity (density) levels in the Basin and in 3 of the remaining years the density layer disappeared in between June and August. All of these were excluded from the correlation.

### Arkona Basin salinity predicting Bornholm Basin oxygen and Eastern Baltic cod egg survival (Analysis #3)

To investigate the functional relationship between salinity measurements at 33 m in the Arkona Basin and the oxygen content in the spawning habitat of Eastern Baltic cod, we averaged oxygen concentrations in the Bornholm Basin within two layers. The first one with the range between 50 and 70 m broadly represents the floating range of eggs of older, repeat spawners. The second one (70 to 95m depth) represents the depth of occurrence of eggs spawned by young females [39,40]. We selected from the averaged time series the oxygen values for April as dependent variable. April represents the beginning of the spawning season of cod and analysis #2 showed that the oxygen conditions in the remaining spawning season depend on this initial content. As independent variable we used maximum salinity values at the 33m depth layer in the Arkona Basin between January and March. The monthly mean salinity here represents a measure of intensity of the water inflow from the Kattegat and the time period was identified as most influential by analysis #1. The capabilities of the found relationship for operational forecasting was tested by the method of leave one out cross validation.

Furthermore, we used the modelled data on oxygen concentration to calculate the potential egg survival probability in the Bornholm Basin, which is based on a modelled egg survival function obtained from experimental data [13]:

$$y_{o}=100({\left(1-e^{-0.71x}\right)}^{11.63})$$ (1)

where y_o_ is the oxygen-dependent survival probability before hatch and x is the oxygen content in ml l^–1^. The function (r^2^ = 0.94) describes a sigmoid curve with almost total mortality at 2 ml l^–1^ oxygen content, ~50% mortality at 4 ml l^–1^, and <10% mortality at 7 ml l^–1^.

To take cod stock structure into account, we assembled model data time series on oxygen-related egg survival probability for the same buoyancy levels (1009, 1011, 1013 kg m^-3^) as described above. The derived values for April each year were then used for a correlation with the maximum salinity value in the Arkona Basin at 33 m between January and March similar as the oxygen data from the two coarser depth layers of 50 to 70 and 70 to 95 m before. Also here the capabilities for operational forecasting of the found relationships was tested by leave one out cross validation.

### Exemplary daily interval time series from the measurement station

To give an example of the potential direct applicability of real time measurements transmitted by the observational platform in the Arkona Basin, we finally present as proof of concept records of salinity measurements from the station together with the oxygen concentration data in the Bornholm Basin from the BSIOM. We increased the temporal resolution for this exercise to a daily interval to keep more of the available resolution of the data transmitted from the measurement station and to emphasise on the rapid fluctuations of salinity in the area. We also added monthly means to connect such “real time” daily measurements to the functional relationships developed before. Furthermore we chose a period with a pronounced major Baltic Inflow, as represented by the winter months in 2014/15 to show the propagation of the inflow between the two locations and exemplary applied the functional relationships to predict the oxygen concentration in the Bornholm Basin at the beginning of April and the associated egg survival probabilities for mid-age and young females.

## Results

**Fig 3:**
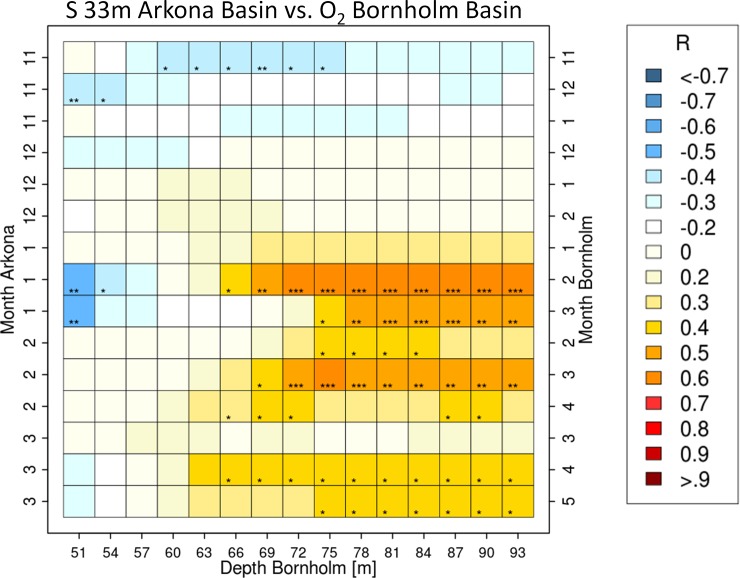
Correlation coefficients of salinity at 33m depth for different months in the Arkona Basin vs. oxygen concentration at various depths for successive months in the Bornholm Basin. Time series of monthly mean values for the correlations were taken from hydrodynamic model runs (BSIOM) hindcasting the period 1970–2015. From the entire period November to March salinity values at the 33m depth level in the Arkona Basin were correlated respectively to the vertical oxygen distribution in the Bornholm Basin between November and May. The value of the correlation coefficient is depicted in colour code for each combination of months selected for salinity and oxygen values and additionally the corresponding depth layer in the Bornholm Basin. The significance of each correlation is depicted with asterisks as follows: p>0.05 = no asterisk, p<0.05 = *, p<0.01 = **, p<0.001 = ***.

Monthly averaged profiles obtained from hydrodynamic model runs were used to study the long-term variability (1971 to 2015) of the Eastern Baltic cod spawning environment. A close coupling of salinity data from the Arkona Basin with the oxygen concentrations below the halocline in the Bornholm Basin was found by using cross-correlation statistics with detrended time series. Fig 3 shows the cross correlations between salinity at the 33m depth level in the Arkona Basin and the oxygen concentrations in different depths in the Bornholm Basin. Investigated time lags for the oxygen time series in the cross-correlations with the salinity time series in November, December, January and February, were selected as 0,1 and 2 months. Positive correlations are indicative of the chronology and the predominant offset point of an increase in the amount of imported saline, oxygen-rich North Sea water. Salinity values in November and December were found not to be useful in any of the time lags for predictions of oxygen levels in the Bornholm Basin with r < 0.3 and low significant levels. The highest significant correlations were found to occur for the 33m salinity level in the Arkona Basin in January and the oxygen content in the Bornholm Basin at depth levels below 72m in February (Fig 3, 0.5 < r < 0.6; p<0.001). But also salinity in February and oxygen conditions in March showed significant correlations of 0.4 < r < 0.5 for depth layers below 75m. Significant positive cross-correlations were also found for February salinity values and oxygen values in March below 69m (0.4 < r < 0.6; p<0.01). Weaker correlations between salinity values in March and oxygen conditions in April and May were also present with 0.3 < r < 0.4 but less significant (p <0.05).

**Fig 4:**
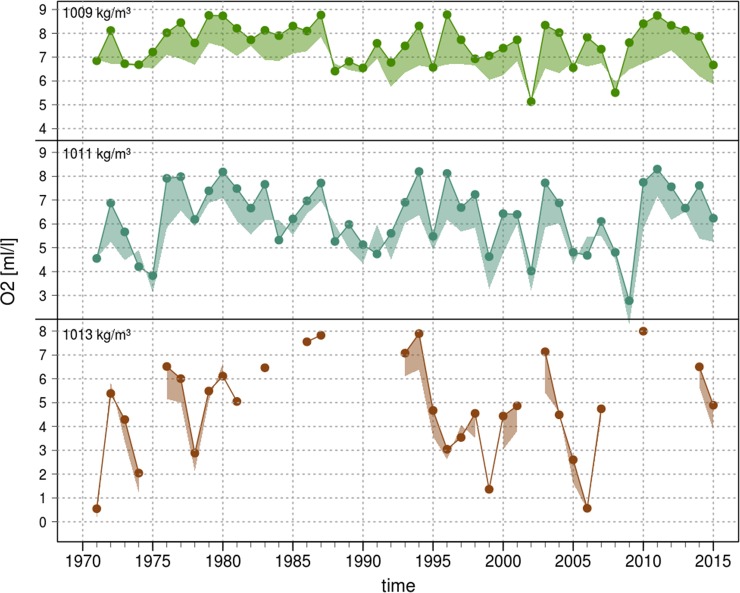
Oxygen concentration time series for the Bornholm Basin on neutral egg buoyancy levels for the period 1971–2015. The density levels represent neutral buoyancy of Eastern Baltic cod eggs spawned by old (1009 kg m^-3^, top panel), middle aged (1011 kg m^-3^, middle panel), or young females (1013 kg m^-3^; bottom panel), respectively. Dots and solid lines represent monthly mean values in April of the years 1971–2015 and shaded areas the differences to the monthly mean values in August of the same year. Data was taken from hydrodynamic model runs (BSIOM).

**Fig 5:**
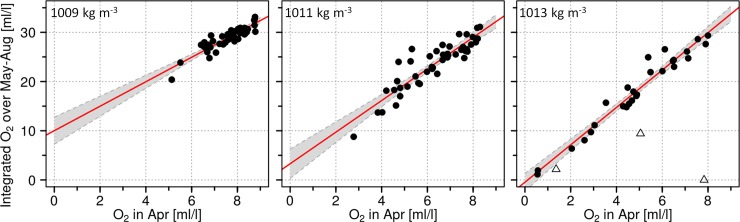
Oxygen content relationships in the Bornholm Basin on neutral egg buoyancy levels between the beginning (April) and the rest of the spawning season (Integral May-Aug). Monthly means of the oxygen content on 3 density levels in the Bornholm Basin derived from hydrodynamic model runs (BSIOM) hindcasting the period from 1971 until 2015 were used for the analysis. The density levels represent neutral buoyancy of eggs spawned by old (1009 kg m^-3^, left panel), middle aged (1011 kg m^-3^, middle panel), and young females (1013 kg m^-3^, right panel), respectively. Linear regression lines are shown in red and the 95% confidence intervals as gray shaded areas. Regression parameters are shown in Table 1. Δ = represents years in which the density layer did not exist for the entire spawning season.

These results demonstrate a correlative link within the inflow season between salinity in the Arkona Basin and oxygen concentration in the Bornholm Basin a few weeks later. Fig 4 shows that there are large interannual variations in the prevailing oxygen conditions in the Bornholm Basin at the beginning of the spawning season but with the common feature of decreasing concentrations from April to August (in 91% of cases). Eggs spawned by old repeat spawners floating around the 1009 kg m^-3^ density level inhabit a less variable habitat in April (5–9 ml/l O_2_; Fig 4 top panel) than the ones spawned by young females which are neutral buoyant at 1013 kg m^-3^ (0.5–8 ml/l O_2_; Fig 4 bottom panel). Regressions between the April oxygen content and the integrated oxygen content over May to August (Fig 5 and Table 1) quantify moreover that the overall oxygen conditions throughout the whole spawning season are depending on the prevailing oxygen level in April. For all the density levels strong correlations were obtained (Adj. R^2^ = 0.48–0.95) with only the estimation of the intercept parameter for the 1013 kg m-3 level showing a none-significant p value of 0.6. Overall these results indicate that the processes determining the spawning season’s oxygen conditions for cod are mostly completed upon April, confirming results from Hinrichsen et al (Fig 7 in [40]). This also justifies the chosen limitation of the analyses to the months November to April.

**Table 1:** Statistical parameters of linear regressions shown in Fig 5.

*Monthly mean oxygen content in the Bornholm Basin [ml/l]*: *April vs*. *Integral over May to August*				
*Density level* ***1009 kg m***^***-3***^ *(Fig 5 left panel):*				
Rsqr	Adj Rsqr	Standard Error of Estimate		
0.8169	0.8127	1.026		
Parameter	Coefficient	Std. Error	t	p
Intercept	10.01	1.3686	7.314	< 0.001
Slope	2.4866	0.1795	13.852	< 0.001
*Density level* ***1011 kg m***^***-3***^ *(Fig 5 middle panel):*				
Rsqr	Adj Rsqr	Standard Error of Estimate		
0.819	0.48148	2.123		
Parameter	Coefficient	Std. Error	t	p
Intercept	3.2948	1.484	2.22	0.0317
Slope	3.2091	0.230	13.95	< 0.001
*Density level* ***1013 kg m***^***-3***^ *(Fig 5 right panel):*				
Rsqr	Adj. Rsqr	Standard Error of Estimate		
0.9534	0.9525	1.725		
Parameter	Coefficient	Std. Error	t	p
Intercept	-0.464	0.8795	-0.528	0.602
Slope	3.7991	0.1662	22.859	<0.0001

**Fig 6:**
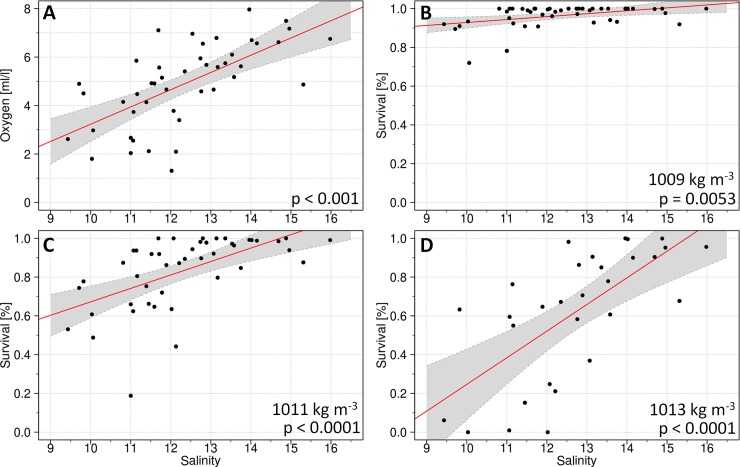
Correlations of monthly mean salinity values in the Arkona Basin at 33m depth vs. oxygen content and oxygen-related egg survival in the Bornholm Basin. The explanatory variable in all panels is the maximum monthly mean salinity value between January and March at the 33m depth level in the Arkona Basin for the years 1971 to 2015. As dependent variables the mean oxygen condition and oxygen related egg survival probabilities in the Bornholm Basin in April of the same year were chosen. Panel A shows the linear correlation to the mean oxygen content in the depth range from 70 to 95m; B, C and D, the correlations to the oxygen-related Eastern Baltic cod egg survival probability at the egg neutral buoyancy level 1009, 1011 and 1013 kg m^-3^, respectively. Linear regression lines are shown in red and the 95% confidence intervals as gray shaded areas.

As a next step, we tested for a functional relationship between salinity in the Arkona Basin at 33m depth and the spawning conditions for Eastern Baltic cod in the Bornholm Basin. In the first part of analysis #3 involving oxygen, we separated two depth layers: the range between 50 and 70 m broadly represents the floating range of eggs of older, repeat spawners; The second one (70 to 95m depth) represents the depth of occurrence of eggs spawned by young females. From both series the mean oxygen content in April was correlated to the maximum of the monthly mean salinity values in the Arkona Basin between January and March. For the upper level no correlation was found, while for the lower level a significant correlation was obtained (Adj. R^2^ = 0.41, p < 0.001; Fig 6A; Table 2). The standard residual error of the overall fit was found to be 1.31 and the cross validation mean test error was calculated as 1.8.

**Table 2:** Statistical parameter and cross validation results of the linear functions shown in Fig 6.

*Maximum Salinity Jan. to Mar. Arkona Basin 33m [psu] vs. oxygen content [ml/l] Apr. Bornholm Basin 70-95m (Fig 6A):*					
Rsqr	Adj Rsqr	Std. error residuals	F	p	d.f.
0.4236	0.4102	1.31	31.6	<0.001	43
Parameter	Coefficient	Std. Error	t	p	
y_0_	-3.8837	1.5741	-2.467	0.0177	
slope	0.7114	0.1266	5.621	<0.0001	
Cross validation results					
Mean Rsqr	Std. deviation	Mean training error	95% conf. int.	Mean test error	95% conf. int.
0.4237	0.0156	1.638	0.1103	1.758	5.023
*Maximum Salinity Jan*. *to Mar*. *Arkona Basin 33m [psu] vs*. *oxygen-related egg survival Apr*. *Bornholm Basin buoyancy level 1009 [kg m*^*-3*^*] (Fig 6B):*					
Rsqr	Adj Rsqr	Std. error Residuals	F	p	d.f.
0.1674	0.148	0.0532	8.643	0.0053	43
Parameter	Coefficient	Std. Error	t	p	
y_0_	0.777	0.064	12.15	<0.0001	
slope	0.015	0.005	2.94	0.0053	
Cross validation results					
Mean Rsqr	Std. deviation	Mean training error	95% conf. int.	Mean test error	95% conf. int.
0.1675	0.0126	0.0027	0.0004	0.003	0.0169
*Maximum Salinity Jan*. *to Mar*. *Arkona Basin 33m [psu] vs*. *oxygen-related egg survival Apr*. *Bornholm Basin buoyancy level 1011 [kg m*^*-3*^*] (Fig 6C):*					
Rsqr	Adj Rsqr	Std. error residuals	F	p	d.f.
0.346	0.331	0.1505	22.75	<0.0001	43
Parameter	Coefficient	Std. Error	t	P	
y_0_	-0.0211	0.1808	-0.117	0.907	
slope	0.0694	0.0145	4.770	<0.0001	
Cross validation results					
Mean Rsqr	Std. deviation	Mean training error	95% conf. int.	Mean test error	95% conf. int.
0.3464	0.0136	0.0216	0.0023	0.0235	0.1054
*Maximum Salinity Jan*. *to Mar*. *Arkona Basin 33m [psu] vs*. *oxygen-related egg survival Apr*. *Bornholm Basin buoyancy level 1013 [kg m*^*-3*^*] (Fig 6D):*					
Rsqr	Adj Rsqr	Std. error residuals	F	p	d.f.
0.459	0.4395	0.2504	23.74	<0.0001	28
Parameter	Coefficient	Std. Error	t	p	
y_0_	-1.1279	0.36143	-3.121	0.0042	
slope	0.1374	0.02821	4.872	<0.0001	
Cross validation results					
Mean Rsqr	Std. deviation	Mean training error	95% conf. int.	Mean test error	95% conf. int.
0.4590	0.0235	0.0584	0.0048	0.0671	0.1480

For the second part of analysis #3 involving oxygen-related egg survival, we further refined the vertical resolution but kept the setup of correlating the values of the dependent variable in April to the maximum of the monthly mean salinity level in the Arkona Basin at 33m between January and March. Survival probability was calculated at three buoyancy/density levels of 1009, 1011 and 1013 kg m^-3^ representing levels of neutral buoyancy of eggs spawned by old, mid-age and young females, respectively. The results for the density layers showed significant differences (Fig 6; Table 2). Survival of eggs spawned by old females were almost independent on the 33m salinity level in the Arkona Basin with an adj. R^2^ of 0.15, while the survival probability for eggs spawned by mid-age and young females could be related to the salinity level in the Arkona Basin with adj. R^2^ of 0.33 and 0.44, respectively. These correlations were also found highly significant with p values < 0.0001 on 43 and 28 degrees of freedom. The mean test errors of the performed leave one out cross validation resulted in 0.024 and 0.067; and the residuals of the overall fit to the data had a standard error of 0.053 and 0.151 for the 1011 and 1013 kg m^-3^ density level, respectively.

**Fig 7:**
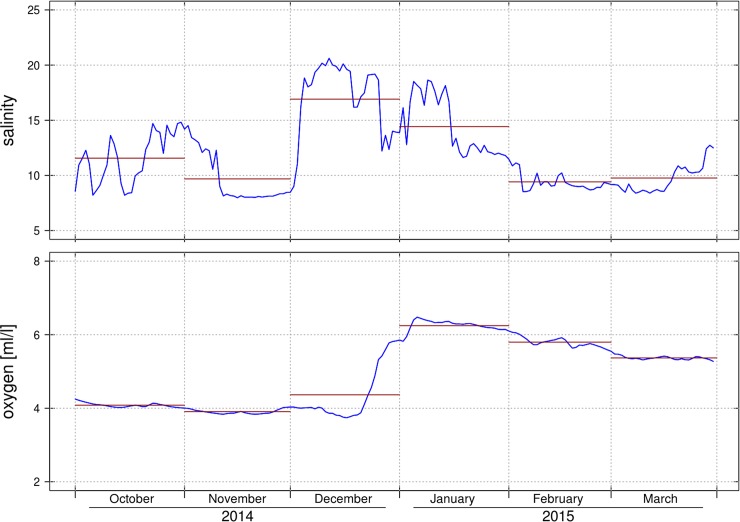
Comparison of measured salinity at 33m from the Arkona Basin and oxygen content in the Bornholm Basin from modeled data. Time series of salinity measured at the 33m depth level (Arkona Basin platform) operated by the Federal Maritime and Hydrography Agency, Hamburg, Germany is shown in daily resolution in the upper panel. Lower panel represents vertically mean oxygen concentration data (averaged between 60 and 90m) of the Bornholm Basin taken from the hydrodynamic model runs (BSIOM). Monthly means are indicated as horizontal lines.

An example to demonstrate how routinely performed measurements in the western Baltic Sea could be utilised for the short-term prediction of the Eastern Baltic cod spawning environment is shown in Fig 7. The combination of salinity data (daily interval and monthly averages) measured at the 33m level in the Arkona Basin with the average oxygen concentration below the halocline in the Bornholm Basin taken from the hydrodynamic model showed, that the major Baltic inflow at the end of 2014 [17] was observed in the Arkona Basin in December (mean S in December = 16.91), while the improvements of the oxygen concentration within and below the halocline (60, 75 and 90m) in the Bornholm Basin occurred around 3 weeks later (mean oxygen in January = 6.24 ml/l). The developed functional relationships, however, do have the requirement to use the maximum of the monthly mean salinity from the Arkona Basin between January and March. Therefore using the monthly mean salinity for January of 14.42 and the relationships presented in Table 2 the oxygen content in the 70-95m depth layer in the Bornholm Basin of 6.37 ± 0.66 ml/l is predicted for April. The data derived from the BSIOM setup resulted in a monthly mean of 5.33 ml/l for this month and water layer. This deviation is well inside of the functional capacity of the relationship tested by cross validation (mean test error of 1.6; Table 2). Furthermore for the spawning season of 2015 the egg survival probabilities for eggs spawned by young and mid-aged females would have been predicted to be exceptionally good with 68–100% and 89–100%, respectively.

## Discussion

In this study we investigated the possibility of predicting the Eastern Baltic cod spawning environment based on salinity measurements from a permanently installed monitoring platform located in the western Baltic Sea. We identified the salinity in the Arkona Basin at 33 m depth level as good short-term indicator for the oxygen conditions in the Bornholm Basin. Although inflow events were recorded frequently also in November and December [21], we identified for the last 40+ years the months January to March as most influential for the oxygen conditions in the Bornholm Basin. Baroclinic inflows during the summer were regularly observed from 1996 on [41] and could become more frequent in the future [1], but our results suggest, that their impact on the spawning habitat of Eastern Baltic cod is not as strong as for major inflows occurring during the winter season. One reason could be the higher temperature of the inflowing water mass associated with less dissolved oxygen. Baroclinic inflows were suspected to extend the habitat for eggs spawned by young females in July [40], but in this study we show that the oxygen conditions in April are very good indicators of the oxygen conditions for the entire spawning season.

Direct prediction of the Eastern Baltic cod spawning environment in terms of egg survival probability from salinity measurements in the Arkona Basin at the 33 m depth level is shown here to be possible for the eggs spawned by mid-age and young females, which currently predominate the stock structure [8]. Based on the functional relationship developed in this study, egg survival probabilities would be available by the end of March each year. Old repeat spawners of the Eastern Baltic cod stock produce such large and buoyant eggs that their survival is not strongly dependent on large inflow events. Their eggs float on top of the vertical layers which could become oxygen depleted during the summer and / or stagnation years and therefore have often superior survival probabilities for their eggs than younger females. This again stresses the fact that large females do have a high value for the stocks survival and productivity [42] and efforts should be made to strengthen the larger length classes in the stock by decreasing their fishing mortality. With that the stock could be less affected by the impact of bad environmental conditions, which reduce, as we show in this study, substantially the survival probabilities of smaller and less buoyant eggs spawned by midsized and small females.

These findings were based on data from the hydrodynamic Kiel Baltic Sea Ice-Ocean Model (BSIOM) [26], since in the preliminary analysis in-situ measurements were found not to be sufficient in resolution and temporal coverage. The relationships developed here were intended to be used with real-time data from the Arkona Basin monitoring platform, which is located in a highly variable abiotic environment, making the use of highly resolved time series unavoidable. It was chosen nevertheless due to its benefits such as being ready to use and imminent data source, which enables utilization in short-term forecasts. The hydrography and oxygen concentrations as simulated by BSIOM are in good agreement with observed variations and dynamics of the system [26]. The incorporated oxygen sub-model uses sub-basin dependent primary production rates, which are further scaled to follow observed long-term primary production development [43]. The applied consumption rates are furthermore depending on temperature and oxygen content. The sub-model extends thereby the approach of constant rates for each sub-basin, which is deemed to be already a good approximation [44]. Therefore the BSIOM is a sufficient data source to replace the in-situ measurements for use in this study and the developed functional relationships could be used in future work on incorporating an environmental factor in the Eastern Baltic cod stock assessment procedure. The relationships might have also been calculated based on coupled physical-biogeochemical models such as ERGOM [45] available for modelling hydrography and oxygen distribution in the Baltic Sea. ERGOM was shown to be a very good tool to investigate the upper mixed layer of the system, but also showed weaknesses especially in the model performance for the deep parts of the Baltic Sea sub-basins [46]. A direct comparison between different numerical model approaches is, however, missing. Moreover, complex coupled physical-biogeochemical models often suffer from high levels of noise in the data, which could in turn weaken the forecasting power of derived functional relationships while the fit does not necessarily benefit from the complexity. Since the subject of this study was to find such relationships with the greatest forecasting power, the BSIOM could be a superior model choice. Nevertheless future work that aims at comparing model approaches and improving their applications for fisheries science would be beneficial.

The International Council for the Exploration of the Seas (ICES) is the main fishery advice giving body for the North-Atlantic region, including the Baltic Sea. ICES specifically acknowledged the need to develop and identify ways forward to include environmental and economic considerations in standard fishery advice [47]. For the case of the Baltic Sea fisheries, a series of working group meetings and workshops (e.g. WGIAB, WKDEICE, DEMO workshops) was held to work on Integrated Ecosystem Assessment (IEA), to be used for advice, management strategy evaluation of Baltic fish stock and science communication [8,48].

The recent history of the Baltic cod fisheries exemplifies the need to proceed on this way, and illustrates the potential value of environmental forecasting, as proposed in this paper. Both Baltic cod fisheries, the western as well as the Eastern cod stock fishery, suffered from dramatic quota reductions. E. g. for the western cod stock a Total Allowable Catch (TAC) reduction of 85% was suggested in 2016 [28]. The Eastern Baltic cod fishery faced similarly hard reductions by 2015 [28]. In both cases, the timely and thoughtful inclusion of environmental information in stock assessment and advice might have attenuated the economic consequences, and maybe even buffered stock decline. Here, the early recognition of adverse environmental conditions for reproduction might serve as an early warning indicator, and hint to setting more precautionary fishing limits. By doing so, a buffer against environmentally-induced reductions in stock productivity might be created.

Here, we show that suitable data are already collected on a regular basis. For short-term predictions the existing observational platform can be used. Periods of increased salinity at the 33m level within the winter months can be identified and with the relationships shown in this study an estimation be made of the seasonal egg survival probability spawned by the predominant size classes within the stock, as presented exemplarily for the inflow year 2014/15. In a similar manner data of particularly low salinities from the Arkona Basin would result in very low egg survival probabilities that could be taken as an early warning indicator for a lowered reproductive success, with its impact on the cod stock structure during the following years. This offers a cost-neutral way of informing stock assessment of Eastern Baltic cod, proceeding on the way to more integrated advice. Advice finding efforts usually start in April / May each year. Our results give the opportunity to include environmental information about the entire following spawning season to this time already, where as monitoring cruises by ship and direct measurements give complete information only in hindsight when assessment calculations are already in an advanced state. As discussed during relevant ICES workshops (e.g. WKDEICE) [48] such information might be used in different ways to inform stock assessment and advice [49]:

1. Using an environmentally-sensitive stock-recruitment function in the short-term forecast of the current standard procedure;
2. Using all relevant information from an environmental assessment to modify fishing mortalities in short-term forecast; depending on the state and trend in environmental conditions, F-multiplier could be used, allowing for increased or decreased fishing opportunities, respectively.
3. Fishing mortality (F)-multipliers could be directly used in the advice giving process, reflecting environmental assessment outcomes.

The utilisation of salinity as relatively simple indicators allows a general examination, how the environmental conditions affect Baltic cod egg development and survival, and is relatively inexpensive compared to field observations. Investigating and deciding, however, how to include proxies of stock productivity into the advice finding process is outside of the scope of this study. Modifications of recruitment models and sections of population analyses within the assessment incorporating our findings and also other possibilities on the way towards an environmental integrated advice need to be subject to future studies.
